# Cutaneous Toll-like Receptor 9 Pre-Defines Hydroxychloroquine Dosage in Patients with Both Discoid and Subacute Lupus Erythematosus

**DOI:** 10.3390/medicina59112022

**Published:** 2023-11-17

**Authors:** Karolina A. Englert, Grzegorz Dyduch, Agata Kłosowicz, Magdalena Spałkowska, Andrzej Kazimierz Jaworek, Kamila Migacz-Gruszka, Aleksandra Jarosz-Chudek, Santo Raffaele Mercuri, Joanna Szpor, Gianluigi Mazzoccoli, Giovanni Damiani, Anna Wojas-Pelc

**Affiliations:** 1 Department of Dermatology, University Hospital in Krakow, 31-501 Kraków, Poland; englertderm@gmail.com (K.A.E.); agata.klosowicz@uj.edu.pl (A.K.); mspalkowska@gmail.com (M.S.); andrzej.jaworek@uj.edu.pl (A.K.J.); kamila.migacz@uj.edu.pl (K.M.-G.); a.jarosz987@interia.eu (A.J.-C.); anna.wojas-pelc@uj.edu.pl (A.W.-P.); 2 Department of Pathomorphology, Jagiellonian University Medical College in Krakow, 33-332 Kraków, Poland; grzegorzdyduch@gmail.com (G.D.); joanna.szapor@uj.edu.pl (J.S.); 3 Unit of Dermatology, IRCCS San Raffaele Hospital, 20132 Milan, Italy; mercuri.santoraffaele@hsr.it; 4 Italian Center of Precision Medicine and Chronic Inflammation, 20122 Milan, Italy; 5 Division of Internal Medicine and Chronobiology Laboratory, Department of Medical Sciences, Fondazione IRCCS “Casa Sollievo della Sofferenza”, 71013 San Giovanni Rotondo, Italy; 6 Department of Biomedical, Surgical and Dental Sciences, University of Milan, 20122 Milan, Italy

**Keywords:** cutaneous lupus erythematosus, discoid lupus erythematosus (DLE), subacute cutaneous lupus erythematosus (SCLE), CLASI, hydroxychloroquine response, precision medicine, Toll-like receptors type 9 (TLR-9)

## Abstract

*Background and Objectives*: Cutaneous lupus erythematosus (CLE) presents clinically heterogeneous manifestations, partially explained by the different expression of Toll-like receptors (TLRs) type 8 and 9, located to endosomal compartments where they are poised to recognize microbial nucleic acids. This disease is empirically treated with hydroxychloroquine (HCQ), which is hallmarked with a safe and effective profile, but induces a slow and sometimes clinically insufficient therapeutic response. Currently, no biomarkers predictive of response are validated or even proposed in the scientific literature. We aimed to evaluate endosomal TLR type 7, 8 and 9 as predictive biomarkers of HCQ efficacy. *Materials and Methods*: We conducted a case–control study comparing CLE patients retrospectively assigned to three subgroups based on 3–6-month Cutaneous LE Disease Area and Severity Index (CLASI) reduction upon treatment with HCQ (I = <40% vs. II *=* 40–80% vs. III = >80%). Before HCQ, lesional skin specimens were collected in untreated CLE and through immunohistochemistry; TLR-7, -8 and -9 expression was evaluated in the epidermis and the lymphocytic infiltrate was evaluated in the dermis. *Results*: Sixty-six lesional skin biopsies were compared with healthy controls. CLE patients displayed lower epidermal expression of total TLR 8 and 9 as well as infiltrating TLR-8, TLR9 + lymphocytes compared to controls. High HCQ responders differed from low responders for TLR-9 positivity (high vs. low) and for the lymphocytic dermal infiltrate (high vs. low). *Conclusions*: TLR9 could be envisaged as a possible biomarker to predict HCQ response level and dosage in CLE patients.

## 1. Introduction

Cutaneous lupus erythematosus (CLE) encompasses several clinical entities ranging from discoid lupus erythematosus (DLE) to subacute cutaneous lupus erythematosus (SCLE), potentially affecting both skin and mucosa. Despite its pathogenesis remaining not fully clear, a common histopathological figure shared by all CLE manifestations is the “interface dermatitis” pattern together with dermo-epidermal deposits of both autoantibodies and a complement [1].

An inflammatory infiltrate is widely represented and purely composed of Th-1 lymphocytes and plasmacytoid dendritic cells producing the type I interferon (IFN). Type I IFN controls both innate and adaptive immunity and regulates the intracellular antimicrobial programmed response, altering exposome perception (i.e., antigen presentation) and cutaneous microbiome homeostasis [2]. This results in cutaneous dysbiosis, increased photosensitivity, inflammation and consequent tissue damage. Damage-derived self-DNA may erroneously trigger autoimmunity in susceptible patients and can cause CLE [1,2,3,4].

The hypothesis of dysbiosis as *primum movens* in CLE is debated, but Toll-like receptor (TLR)-7, -8 and -9, a class of pattern recognition receptors (PRRs) capable of sensing microbes also in the skin, is presently regarded as crucial in the susceptibility [5], promotion and maintenance of systemic lupus erythematosus (SLE) in both mouse models [6] and patients [6,7,8,9,10,11]. Recently, a potential role of TLRs was postulated also in CLE [7,8] and randomized clinical trials started to test innovative biological drugs targeting TLRs [9].

**Figure 1:**
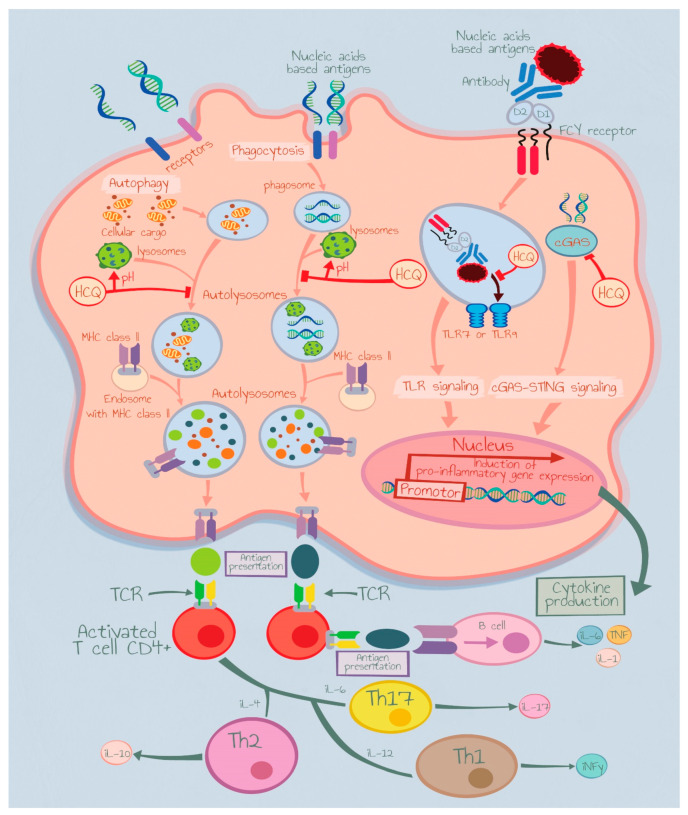
Hydroxychloroquine mechanism of action. IL: Interleukin, TCR: Toll-Like Receptor, Th: T Helper Cells.

Interestingly, the inexpensive antimalarial drug hydroxychloroquine (HCQ), first-line treatment for CLE with topical corticosteroids, calcineurin inhibitors, has effective immunomodulatory action and may block the signaling cascade prompted with TLR-7 and -9 [10] (Figure 1). Despite the wide use of this drug, CLE patients show heterogeneous responses and unfortunately no predictive biomarker is available at present in clinical practice.

Since a CLE diagnosis always deserves anatomopathological confirmation, a clinical study to evaluate TLR-7, -8 and -9 presence in CLE lesional skin specimens and their potential use as predictive biomarkers of response to HCQs was designed.

## 2. Materials and Methods

### 2.1. Ethics

This study complies with the Declaration of Helsinki and was performed according to ethics committee approval. The protocol was approved by the Bioethical Committee of Jagiellonian University, Krakow, Poland (protocol number: 1072.6120.48.2019, 28 February 2019). All patients signed an informed consent form.

### 2.2. Study Design

In this case–control study, skin biopsies of untreated CLE (SCLE and DLE) patients were compared with healthy controls, assessing the prevalence of TLR-7, -8 and -9 expression. All samples were obtained from the Pathomorphology Department of Jagiellonian University Hospital in Krakow, Poland. Control samples were collected from lozenge unaffected tails collected from the buttock area, a typical UV protected area. Besides the surgical extraction, controls enrolled were adults (>18 years old) and apparently healthy without recent history of tumors (>5 years).

CLE patients were then subdivided into three groups based on HCQ response:

(1) patients treated with daily 200 mg of HCQ;
(2) patients treated with daily 400 mg of HCQ due to an insufficient response to daily 200 mg of HCQ;
(3) patients treated once with topical high-power corticosteroids (i.e., clobetasol).

Adult CLE cases were enrolled if they (1) displayed SCLE and DLE clinical phenotypes, (2) had only CLE, (3) were treated only with HCQ in monotherapy or topical steroids monotherapy, (4) had a skin biopsy before starting any treatments (both topical and systemic ones) and (5) signed informed consent for specimen scientific re-evaluation.

The exclusion criteria for subjects’ enrollment included (1) HCQ treatment lasting less than 3 months; (2) skin biopsy performed after the initiation of systemic immunomodulatory therapy; (3) treatment with other immunosuppressive medications such as methotrexate and mycophenolate mofetil, (4) a diagnosis of multiple chemical sensitivity [11] or atopic dermatitis [12] and (5) refusal to sign informed consent.

### 2.3. Clinical Evaluation

The severity of skin lesions was assessed using the CLASI index [13] in the study group, before the initiation of systemic immunomodulatory therapy and after 3–6-month treatment. Then, the enrolled patients were divided into 3 subgroups depending on the percentage of decrease in the CLASI index after the 3–6-month treatment. The subgroups were as follows:

- Subgroup 1—patients with reduction in the CLASI index after the 3–6-month treatment (<40%).
- Subgroup 2—patients with reduction in the CLASI index after the 3–6-month treatment ranging from 40 to 80%.
- Subgroup 3—patients with reduction in the CLASI index after 3–6-month treatment (>80%).

Photographic images of obtained results were captured using the Olympus CX 40 microscope equipped with an Olympus DP 70 camera (Olympus; Tokyo, Japan).

### 2.4. Staining Protocol

Cutaneous tissue samples were stained manually and processed according to the protocol used on a routine basis in the laboratory of the Department of Pathomorphology, Jagiellonian University, Krakow, Poland. The selected paraffin-embedded tissue sections, 3- to 4-µm-thick, were mounted on SuperFrost glass slides (ThermoScientific, Waltham, MA, USA) and dried in an incubator for 12 h at 34 °C. Three slides from each cutaneous biopsy were obtained and then divided into 3 equal sets. Subsequently, the slides were deparaffinized, dehydrated and then incubated in a 3% H_2_O_2_ solution for 10 min to block endogenous peroxidase activity. Antigen retrieval was performed by immersing the slides in EDTA (pH 8.0; 0.01 M) and subjecting them to 97 °C in a water bath for 30 min. Each set of slides was then incubated with specific anti–TLRAbs for 12 h. The first set of slides was incubated with the rabbit monoclonal anti-TLR7 antibody (EPR2088(2); Abcam (Cambridge, UK); dilution of 1:100); the second set of slides was incubated with the mouse monoclonal anti-TLR8 antibody (44C143; Abcam; dilution of 1:100); the third set of slides was incubated with the mouse monoclonal anti-TLR9 antibody (26C593.2; Abcam; dilution of 1:100). Polyclonal secondary antibodies conjugated to the horseradish peroxidase (HRP) enzyme (Ultra Vision LP Value Detection System HRP Polymer, Lab Vision, ThermoScientific, USA) were applied to visualize the obtained antigen–antibody complexes, using DAB (3,3′-diaminobenzidine) as the chromogen. Finally, cell nuclei were stained with hematoxylin to enhance contrast in tissue sections.

### 2.5. Histopathological Evaluation

Stained biopsy sections were independently evaluated by two board-certified pathologists (G.D.; J.S.) with a semi-quantitative method. TLR-7, -8 and -9 were evaluated in the epidermis and in the dermal lymphocytic infiltrate using intensity and distribution scores.

The intensity score ranged from 0 (absence) to 3 points (strong positivity); conversely, the distribution score evaluated the epidermal thickness percentage of immunohistochemical positivity to the TLRs together with the percentage of infiltrating lymphocytes per follicle with TLR-positive expression. Moreover, the thickness of the epithelial part of hair follicles with positive immunohistochemical reactions for type 7, 8 and 9 TLRs was also assessed and presented as a percentage value (0–100%).

### 2.6. Statistical Analysis

The statistical analysis was performed using The R 4.0.5 project (R Core Team 2021, R: a language and environment for statistical computing. R Foundation for Statistical Computing, Vienna, Austria. URL: https://www.R-project.org/). To compare TLR expression between CLE patients and the control group, the Fisher exact test or the Wilcoxon rank-sum test for nonparametric data were used. In the statistical analysis of TLR expression in subgroups of SCLE and DLE patients and the control group, the Fisher exact test or Kruskal–Wallis tests for nonparametric data were used. To evaluate TLR9 expression in groups according to the reduction in the CLASI index in CLE patients treated with HCQ, the Fisher exact test or Kruskal–Wallis tests for nonparametric data were used. TLR9 expression in groups according to the need of an HCQ dosage increase from 200 mg to 400 mg was analyzed by using the Fisher exact test or the Wilcoxon rank-sum test for nonparametric data. Values of *p* < 0.05 were considered significant.

## 3. Results

Lesioned skin samples were obtained from 66 patients diagnosed with DLE (*n* = 28) or SCLE (*n* = 38). There were 47 females and 19 males with a mean age of 53.3 years (Table 1).

**Figure 2:**
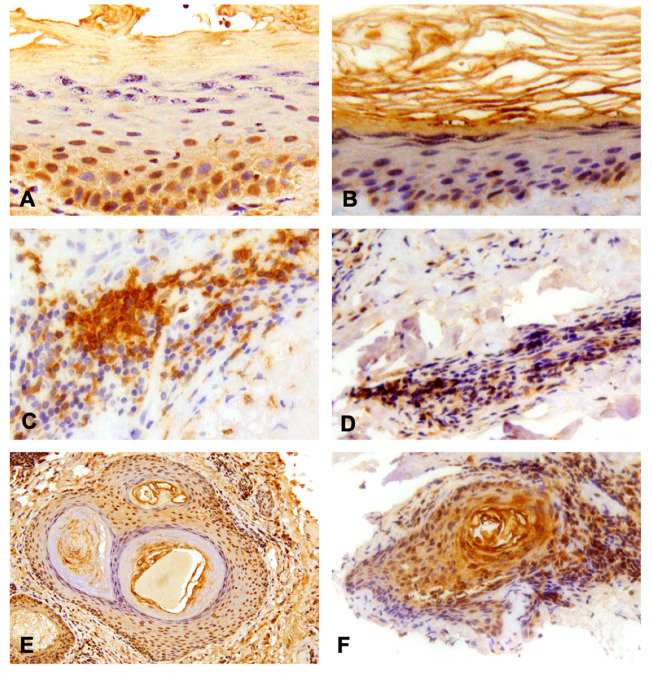
Results of the immunohistochemistry analysis. (**A**) Positive expression of TLR-8 in granular layer of the epidermis, 40× magnification; (**B**) Positive expression of TLR-9 in the spinal layer of the epidermis and partially in the granular one, 20× magnification; (**C**) Dermal infiltration of lymphocyte TLR-8 (intensively positive), 40× magnification; (**D**) Dermal infiltration of lymphocyte TLR-9 (mildly positive), 20× magnification; (**E**) TLR-8 and (**F**) -9 expression in the context of hair follicles, 10× and 20× magnification respectively.

In the analyzed samples, there was no evidence of TLR-7 expression in the epidermis of both CLE patients and controls. Skin biopsies of both healthy skin and CLE lesions showed the expressions of type 8 and 9 TLR in the epidermis and epithelial part of hair follicles and expressions of TLR-7, -8 and -9 in the lymphocytic infiltrate in the dermis (Figure 2).

Patients with CLE showed statistically significantly lower intensity scores of TLR-9 expression in the epidermis (*p* = 0.044), lesser thickness of the epidermis with positive expressions of TLR-8 and -9 (*p* = 0.0033 and *p* = 0.006, respectively) and lower average percentages of lymphocytes with positive expression of TLR-8 and TLR-9 (*p* = 0.030 and *p* = 0.022, respectively), compared to the control group.

The thickness of the epithelial part of hair follicles with positive expression of TLR-9 was lower in SCLE, as well as in DLE patients, compared to the control group (*p* = 0.04). Moreover, patients with SCLE showed significantly higher expression of TLR-8 in the epidermis than patients with DLE (*p* = 0.006). On the other hand, in patients with DLE, a significantly higher expression of TLR-9 in the epidermis and epithelial part of hair follicles was observed compared to the patients with SCLE (*p* = 0.02 and *p* = 0.04, respectively). All of the detailed data are included in the Supplementary Material.

The intensity score of TLR-9 expression in lymphocytic infiltrates in the dermis in patients with cutaneous lupus erythematosus correlated with the response to HCQ treatment and the percentage reduction in the CLASI index after treatment.

Patients with the best response to HCQ treatment (decrease in CLASI index after treatment above 80%) showed the highest TLR-9 expression within the lymphocytic infiltrate in the dermis (3+ in the intensity score) (*p* = 0.029). Moreover, patients whose dose of HCQ was up-titrated to 400 mg, due to the insufficient response to treatment with HCQ at the dose of 200 mg, presented significantly lower expression of TLR-9 in the epidermis (characterized by a smaller thickness of the epidermis with positive expression of type 9 TLR) (*p* = 0.015) and lower average percentages of lymphocytes with positive TLR-9 expression compared to other patients treated with 200 mg of HCQ (*p* = 0.021).

During 2 years of follow up, the patients in our study group did not experience any side effects from HCQ treatment.

## 4. Discussion

The results of our study show a high-density presence of epidermal TLR-9 as well as a TLR-9+ lymphocytic infiltrate that are predictive of the response to HCQ treatment. High expression of cutaneous TLR-9 is typical of CLE and may predict a high response to low-dose HCQ (200 mg per day). To our knowledge, this is the first study addressing the role of the TLR-7, -8 and -9 expression in the pathogenesis of cutaneous lupus erythematosus in humans. As evidenced in murine models, TLR-7 overexpression induces systemic autoimmunity in mouse strains, not prone to develop spontaneously systemic lupus erythematosus [14,15]. In turn, knock-out of TLR-7 restrains lupus erythematosus evolution in mice spontaneously developing this disease [16,17]. Mice prone to develop lupus erythematosus with TLR-7 deletion in B cells were characterized by diminution of autoantibodies against RNA-containing antigens; inefficient class switch recombination of antibodies targeting apoptotic and phospholipid epitopes; decreased number of immune-competent cells, i.e., germinal-center B cells, follicular helper T lymphocytes (TFH), macrophages and neutrophils; and lower disease activity including no development of glomerulonephritis, compared to mice strains with positive expression of TLR-7 [18]. In turn, TLR-8 and TLR-9 seem to restrict the negative role of TLR-7 in the pathogenesis of lupus erythematosus. Desnues B. et al. showed that the course of glomerulonephritis was more severe in TLR-8- and TLR-9-deficient mice, compared to the control group. Additionally, TLR-8- and TLR-9-negative mice were characterized by a higher number of antigen-producing cells and higher autoantibody (ANA) titers, compared to TLR-8- and TLR-9-positive strains. In line with literature evidence, the course of disease in mice strains with double knock-out of TLR-8 and TLR 9 was more severe than in the case of TLR-8 or TLR-9 single knock-out mice [19].

In our study, we showed that higher expressions of TLR-8 and TLR-9 in the epidermis and within the lymphocytic infiltrate in the dermis may play a protective role in the development of cutaneous lupus erythematosus. It was also demonstrated that lower TLR-9 expression within the lymphocytic infiltrate in the dermis and lower expression of TLR-9 in the epidermis may predict lower responsiveness to HCQ treatment in CLE patients.

Current scientific reports concerning endosomal TLR type 7, 8 and 9 expression in keratinocytes are not univocal. Lebre M. et al. found mRNA expressions of TLR type 2, 3, 4, 5, 6, 9 and 10 but not TLR-7 and -8 in cultured human keratinocytes [20]. In turn, Flacher V. et al. revealed positive mRNA expressions of TLR type 7, 8 and 9 in human keratinocytes with ∼10 times more mRNA expression for TLR-7 receptors compared to Langerhans cells. Albeit the authors considered the low levels of TLR-8 expressed by keratinocytes as nonsignificant and the low levels of TLR-9 expressed by keratinocytes as unresponsive to CpG oligodeoxynucleotides [21].

In our study, TLR-7 expression was not observed in skin biopsies except for the lymphocytic infiltrate, which is unexpected since type 7 TLR may contribute to the development of cutaneous lupus erythematosus lesions. Li Z. et al. showed expression of TLR-7 in keratinocytes after calcium-induced differentiation. The investigators proved that TLR-7 is essential for boosting the immunological response such as the activation of NF-κB-dependent signaling and proinflammatory cytokines (TNF-α; IL-8) after imiquimod treatment. They also demonstrated imiquimod-induced expression of TLR7 and proinflammatory cytokines, synergistically with calcium [22].

Conversely, the constitutive expressions of TLR-8 as well as TLR-9 in keratinocytes of the epidermis, in the lymphocytes of the inflammatory infiltrate and in the epithelial part of hair follicles were confirmed. A statistically significant correlation between the level of TLR-8 and TLR-9 expression and the response to HCQ treatment was also demonstrated.

Mande P. et al. showed a negative regulatory role for TLR-9 in the development of systemic autoimmunity in the inducible rapid-onset murine model. The investigators showed that the TLR-9-deficient, autoimmune-prone mice strains developed skin lesions histologically similar to those of CLE in humans. The biopsies of skin lesions were characterized by epidermal hyperplasia, perivascular/perifollicular mononuclear infiltrate, follicular plugging, basal layer vacuolation, minor basement membrane thickening, dermal mucin accumulation, apoptotic cell death in the epidermis and deposits of antibodies at the dermo-epidermal junction [7]. However, it is important to emphasize that there are some differences between the human CLE and murine lupus-like skin inflammation model. The epidermal basement layer in mice is thinner than in humans and constantly thickens in response to inflammation. Therefore, instead of atrophy, typical for humans’ histological CLE changes, the TLR-9-deficient mice developed epidermal hyperplasia, like the inflammatory skin disease that occurs in SLE-prone MRL/lpr mice and the hypertrophic form of cutaneous lupus erythematosus [7].

Moreover, in the study conducted by Mande P. et al., the mice strains that were TLR-9-sufficient or TLR-7-deficient or doubly deficient in TLR-7 and TLR-9 showed no signs of skin inflammation [7]. Similarly, the negative regulatory role of TLR-9 in the development of cutaneous lupus erythematosus was shown, suggesting that lower expression of TLR-8 and TLR-9 in the epidermis and in the lymphocytic infiltrate in the dermis and lower expression of TLR-9 in the epithelial part of hair follicles in CLE patients, compared to healthy skin, could positively affect the development of this disease. Our results are consistent with the SLE studies on animal models and in vitro models, in which high expression of TLR-8 and TLR-9 plays a protective role in the development of this disease, as mentioned above.

The exact mechanism of action of antimalarial drugs (chloroquine, HCQ) is not fully clear. At the molecular level, they modify lysosomal activity, autophagy and various signaling pathways. HCQ may interfere with the processing of TLR-7 and TLR-9 by changing the endosomal pH and consequently inhibiting TLR activation upon extracellular stimuli. The other postulated mechanism of action of HCQ and chloroquine is the inhibition of TLR–ligand interactions by directly binding to nucleic acids at the intracellular level. In addition, chloroquine can hinder TLR-7 signaling stimulated by RNA antigens [23]. In the presented study, it was demonstrated that TLR9 expression in skin biopsies in patients with CLE predicts a good response to HCQ treatment. These findings suggest that HCQ might act with the stimulation of signaling pathways boosted by TLR-9. We hypothesize that TLR-9 constitutes a biological target of HCQ action. Consequently, this drug could be more effective in patients with higher TLR-9 expression within the lymphocytic infiltrate in the dermis, and patients with higher levels of TLR-9 expression in the epidermis and within the lymphocytic infiltrate in the dermis require lower doses of HCQ to achieve clinical remission.

Overall, interference with TLR activation may constitute a promising treatment method in inflammatory autoimmune diseases such as systemic and cutaneous lupus erythematosus. An inhibitor of interleukin-1 receptor-associated kinase 4 (IRAK4) was demonstrated to affect the signaling cascade triggered by TLR-7 and TLR-9 activation and was proved to be more effective in suppressing proinflammatory pathways compared to HCQ [10,24]. Preliminary clinical trials with a selective IRAK4 inhibitor—PF-06650833— showed a favorable safety and pharmacokinetic profile and reduction in inflammatory markers in healthy individuals (30). An alternative strategy for suppressing the inflammatory environment in SLE and CLE may involve boosting TLR signaling. In this context, TLR agonists would act as molecules promoting programmed cell death and enhancing immune surveillance [25].

This study provides new insights into the pathophysiology of cutaneous lupus erythematosus and the mechanisms of HCQ action in this disease entity. However, it does have some limitations, such as a relatively small sample size of patients with CLE from a single dermatology center in Poland and the lack of an independent validation cohort. External validation is necessary to increase the generalizability of the model in a real-world setting. Higher degrees of external validity will be guaranteed by multiple dermatology centers’ participation in the study to provide wider population diversity. In future studies, it would also be beneficial to assess the levels of TLR-9 expression in different forms of cutaneous lupus erythematosus such as acute cutaneous lupus erythematosus and other forms of chronic cutaneous lupus erythematosus such as hypertrophic discoid lupus erythematosus and lupus erythematosus panniculitis. The correlation of TLR-9 expression and the presence of other skin-related symptoms such as non-scarring alopecia and mucosal lesions may also be interesting.

TLR-9 is a multidimensional innate immune receptor located intracellularly and capable of sensing hypomethylated CpG DNA motifs. These motifs are abundant in DNA of a viral or bacterial origin and TLR-9 can both enhance and moderate the inflammatory response, helping to maintain the balance of the body’s defense reaction to pathogens [26,27]. A family of proteins conserved throughout evolution allows the recognition of pathogen-associated molecular patterns (PAMPs). In turn, pattern recognition receptors (PRRs) expressed in innate immune cells such as macrophages and dendritic cells can recognize microbial molecules and trigger upregulation of costimulatory molecules as well as the production of the type I interferon and inflammatory cytokines, leading to maturation of dendritic cells, the efficient presentation of antigens and the establishment of adaptive immunity. TLR9 is unique among all the different PRRs described so far, as it is capable of recognizing bacterial and viral DNA, which drives the TLR9 activation sequence independently, and in turn daily variations in TLR9 expression and function correlate with progression and severity of the inflammatory/immune response [26,27].

The overall host immune response depends crucially on the recognition of microbial infection by innate immune cells. Similarly, the ability of inflammatory/immune cells to detect the presence of the invading pathogen significantly determines the effectiveness of the host’s immune response against infectious agents [26,27].

Interestingly, the expression of TLR9 is characterized by rhythmic oscillations with a periodicity of approximately 24 h, classically called circadian (circa, approximately, and dies, a day) [28]. Living beings exhibit rhythmic fluctuations in cellular processes and tissue functions that are crucial for maintaining the organism’s homeostasis. The rhythmicity of biological phenomena is linked to the presence in all eukaryotic cells of a molecular mechanism that allows for anticipating predictable variations in the external environment, and which provides a selective advantage to organisms and living species that are equipped with it [29]. The rhythmicity of the circadian molecular clocks of living beings is synchronized with environmental cues, primarily light/darkness alternation due to terrestrial rotation. The molecular clockwork functions via intermingled cycles of transcription–translation feedback driven by the expression of so-called clock genes [30].

As abovementioned, the circadian molecular clock controls the rhythmicity of TLR9 expression and function and consequently influences disease severity depending on the timing of receptor activation, which in turn depends on circadian changes in TLR9 protein levels and response intensity. These findings reveal a direct molecular link between the circadian and innate immune systems, with important implications for immune prophylaxis and immune therapy.

## 5. Conclusions

TLR-9 may be a predictive biomarker of the low-dose HCQ response in CLE patients. These data also underline the knowledge gap existing in establishing the pathogenetic role of TLRs in CLE. Furthermore, they also highlight the biological difference existing between CLE patients that rapidly translated into a differential HCQ response. TLR-9 positivity in skin biopsies will be beneficial for the upcoming precision-medicine-based approach also in CLE, allowing clinicians to depict endotypes based on a set of predictive, inexpensive, easy-to-use biomarkers.

Overall, recent scientific data suggest that patients suffering from immune-based diseases could benefit from receiving immunomodulatory therapies during the hours in which they are most susceptible, in order to reduce the necessary dosages of the drug while maintaining its effectiveness but reducing possible side effects. As an interesting perspective, the study of how these factors influence TLR9 expression levels and innate immune responses will be important to prepare therapeutic strategies that can improve the response to therapy and the quality of life of these patients.

## Figures and Tables

**Table 1:** Epidemiologic characteristics of the study population.

	SCLE	DLE	Control
Age (SD)	55.18 (14.86)	49.96 (14.97)	50.34 (10.47)
Sex			
Female, *n* (%)	33 (50.00%)	14 (21.21%)	30 (49.18%)
Male, *n* (%)	5 (7.58%)	14 (21.21%)	31 (50.82%)
Biopsy Location:			
Scalp, *n* (%)	0 (0)	3 (10.71%)	3 (4.92%)
Forehead, *n* (%)	0 (0)	5 (17.86%)	5 (8.20%)
Nose, *n* (%)	0 (0)	3 (10.71%)	3 (4.92%)
Cheek, *n* (%)	0 (0)	9 (32.14%)	9 (14.75%)
Neck, *n* (%)	3 (7.89%)	3 (10.71%)	5 (8.20%)
Chest, *n* (%)	14 (36.84%)	0 (0)	13 (21.31%)
Back, *n* (%)	10 (26.32%)	0 (0)	9 (14.75%)
Arm, *n* (%)	7 (18.42%)	4 (14.28%)	9 (14.75%)
Hand, *n* (%)	2 (5.26%)	1 (3.57%)	3 (4.92%)
Abdomen, *n* (%)	2 (5.26%)	0 (0)	2 (3.28%)
Buttock, *n* (%)	0 (0)	0 (0)	0 (0)

SCLE—subacute cutaneous lupus erythematosus, DLE—discoid lupus erythematosus; LE—the patients with SCLE or DLE treated with HCQ; HCQ—hydroxychloroquine.

## Data Availability

The data presented in this study are available on request from the corresponding author.

## Supplementary Materials

The following supporting information can be downloaded at: https://www.mdpi.com/article/10.3390/medicina59112022/s1, File S1: Clinical and Laboratoristic database.
